# E-commerce Policy and the Global Economy: A Path to More Inclusive Development?

**DOI:** 10.1007/s11575-022-00490-1

**Published:** 2022-11-03

**Authors:** Alan A. Ahi, Noemi Sinkovics, Rudolf R. Sinkovics

**Affiliations:** 1 Business School, King’s College, University of Aberdeen, Aberdeen, AB24 3FX UK; 2 University of Helsinki, Finland Helsinki University, Viikki Campus, 00790 Helsinki, Finland; 3 Adam Smith Business School, University of Glasgow, West Quadrangle, Gilbert Scott Building, Glasgow, G12 8QQ UK; 4 LUT University, Skinnarilankatu 34, PL 20, 53851 Lappeenranta, Finland

**Keywords:** International E-commerce, Policy, Global economy, Governance system, Inclusive E-commerce, Sustainable development

## Abstract

The advancement of digitalization is gradually transforming the existing structure of the global economy. According to the McKinsey Global Institute, almost all cross-border transactions had a digital component in 2016. This is also reflected by the growing literature on digitalization and E-commerce. Yet, studies specifically focusing on E-commerce policy are scarce compared with other areas in this domain. By going beyond academic articles and including policy documents in our analysis, this study takes stock of the issues as well as the policy recommendations identified in these publications. Our analysis reveals that to promote an inclusive E-commerce participation, it is imperative to design policies that improve countries’ formal institutions, facilitate the inclusion of less-developed countries in the E-commerce space, and enhance E-commerce adoption by small- and medium-sized enterprises. We highlight the significance of collaboration between and solidarity among governments and other stakeholders.

## Introduction

The advancement of digitalization is increasingly shaping the way firms conduct business within and across borders (Manyika et al. 2016; Katsikeas et al. 2019; Kraus et al. 2019). Examples include the use of digital technologies to aid their internationalization, enhance their productivity, transform existing or create new business models, and improve interactions with and among consumers (Bouncken and Barwinski 2020; Katsikeas et al. 2019; Sinkovics et al. 2013). Therefore, depending on the nature of the industry, firms can use digital technologies to take advantage of new entrepreneurial opportunities (Kraus et al. 2019; Meltzer 2019) and/or link into global value chains (cf. Bouncken and Barwinski 2020; Sinkovics et al. 2019).

Although digitalization is a broad concept and has many facets (cf. Bouncken and Barwinski 2020; Ritter and Pedersen 2020), electronic commerce (E-commerce) is an important example of how businesses can take advantage of digital technologies. E-commerce may be defined as “the sale or purchase of goods or services conducted over computer networks by methods specifically designed for the purpose of receiving or placing orders” (OECD 2019a, 2019b, 2019c, p.14).^1^ The benefits of E-commerce adoption in a cross-border space – depending on the nature of the product – include the possibility to enter international markets without costly investments in physical facilities abroad (Kraemer et al. 2006; Pezderka and Sinkovics 2011), rapid response to demand conditions, and a more cost-effective personalization of offerings to customers worldwide (Gregory et al. 2017; Katsikeas et al. 2019; Kraemer et al. 2006). Because of these potential benefits, E-commerce has become a widespread business phenomenon gradually transforming the traditional business landscape (OECD 2019c). It has penetrated nearly all aspects of socio-economic relations, and concomitantly, has brought potential disruptions to the fundamental structure of how global trade is carried out and regulated (Neeraj 2019).

However, despite its benefits, cross-border E-commerce remains subject to many trade barriers (Gessner and Snodgrass 2015; Gomez-Herrera et al. 2014) and is not devoid of risk (Pezderka and Sinkovics 2010, 2011). For example, data protection and privacy issues are frequently of concern; yet, regulations to alleviate these concerns are still a work in progress (Meltzer 2019; Wolfe 2019). Other challenges in the international E-commerce space can include customs delays, ambiguous return processes, insufficient transparency on delivery and pricing, and a limited ability to change delivery times and locations (Gomez-Herrera et al. 2014; UNCTAD 2015). Further, cross-border E-commerce may expose firms to various political, legal, and security risks (Grant et al. 2014; Jean et al. 2020; Pezderka and Sinkovics 2011). As a result, not all firms have equal opportunities to engage in E-commerce. Evidence shows that small and medium-sized enterprises (SMEs) lag behind larger firms in adopting E-commerce (OECD 2019c). In addition, there is a major gap between developed and less-developed countries in E-commerce adoption (OECD/WTO 2017).

Many of these risks and gaps originate from underdeveloped formal institutions (Doh et al. 2017; Jean et al. 2020). Especially important in this context is the existence of laws and regulations and the quality of their enforcement (cf. Clegg 2019). Formal institutions are subject to change via public policy developed by the government or its agencies (Clegg 2019). Appropriate government policies generally lead to transparent institutions that can support E-commerce activities and lower the risks of partaking in them (OECD 2019c). In contrast, weak policies underpinned by inefficient legal and regulatory enforcement can hamper economic activity (Doh et al. 2017; Sheng et al. 2011), and augment the risks associated with E-commerce (Jean et al. 2020). As a consequence, many governments have recognized the importance of policy frameworks as a driver of E-commerce participation (UN 2019), as is reflected by E-commerce becoming a top priority for policy-makers at the national, regional and global level since the mid-1990s (OECD 2019c).

However, there remains large variation in E-commerce policies across countries (Fefer 2020). The world regulatory governance system has been slow to adjust its multilateral rule architecture to these modern business realities (Janow and Mavroidis 2019). At the same time, there is a paucity of academic research on public policy related to cross-border E-commerce, with the majority of existing studies adopting a firm perspective. However, digital transformation is not driven only by private decision-making; the role of government policy plays a pivotal role in this process (cf. Clegg 2019).

To this end, this study aims to take stock of the existing knowledge on E-commerce policy scattered across the academic and policy domains. Going beyond the analysis of academic publications is necessary as they are few in number and relatively dated. Therefore, to close the knowledge gap on E-commerce policy in academic publications, we extend the analysis to documents published by international bodies such as the World Trade Organization (WTO) and the Organisation for Economic Co-operation and Development (OECD). We conclude the study by suggesting four areas for future research, with the intention of bringing greater focus to E-commerce policy matters in international business research.

## Types of E-commerce

Through E-commerce, a range of commercial relationships can occur, involving any possible pairing of consumers, businesses or governments (Laudon and Traver 2018; OECD 2019c). The largest type in terms of monetary transaction is Business-to-Business (B2B) E-commerce, where one business focuses on selling to another. Providing online product and service support, communications of company’s products (promotion and advertising), e-procurement of products and services and electronic supply chain management are examples of B2B E-commerce (Gregory et al. 2017). The other type of E-commerce widely discussed in the literature is Business-to-Consumer (B2C) E-commerce, whereby businesses attempt to sell to individual customers. The most prominent business model within B2C E-commerce is online retailers such as Amazon and Alibaba. While traditionally B2B transactions dominated the E-commerce landscape, B2C is rapidly increasing (OECD 2019c). Another major type of E-commerce is Consumer-to-Consumer (C2C) E-commerce, which provides a way for consumers to sell to one another using a platform provided by an online market maker or a platform provider such as e-bay, or on-demand service companies such as Airbnb and Uber (Laudon and Traver 2018). Last, there is business-to-government (B2G) E-commerce, whereby governments are engaged in a commercial relationship with businesses.

While B2B, B2C, C2C, and B2G E-commerce constitute the most common types of E-commerce, other ways to participate in E-commerce have recently emerged that are also growing quickly. For example, mobile E-commerce (m-commerce) is a type of E-commerce through which mobile devices, such as smartphones and tablet computers, are used to complete a commercial online transaction (OECD 2019c). Further, social E-commerce, in which E-commerce is enabled by social networks and online social relationships, is another type of E-commerce that has gained popularity in recent years. A well-known example is Facebook, the leading social network (Laudon and Traver 2018).

These different types of E-commerce share common features (using digital technologies and methods specifically designed to receive or place orders to conduct business). Government policies – for example, regulations regarding consumer protection and data protection and privacy – can affect all types of E-commerce. Therefore, in this paper, we consider policies regarding all types of E-commerce.

## E-commerce Public Policy

Public policies are actions that governments undertake to set goal(s) and utilize means or tools to achieve such goal(s) (Howlett and Cashore 2020). Public policy-making is a dynamic process that is usually the result of a set of interrelated decisions that cumulatively contribute to an outcome (Howlett and Cashore 2014). In an international business context, policy refers to a change governments intentionally make to shape the decision-making and behavior of firms within the international business domain (Clegg 2019; Lundan 2018). Through public policies, governments can decide – within their capacity – whether to act to change or maintain some aspect of the status quo (Birkland 2019; Howlett and Cashore 2014). Governments, for example, can design and implement policies to prioritize the allocation of resources toward the development of a specific sector (Georgallis et al. 2021).

In the context of E-commerce, governments can allocate resources to the development and growth of information and communication technology (ICT) infrastructure and provide access to reliable and affordable ICT services (UNCTAD 2019). This is important as ICT is a key building block of the digital economy, which facilitates and drives E-commerce (OECD/WTO 2017). Through public policy, governments can also design, implement, and communicate up-to-date legal and regulatory frameworks that provide a supportive business environment for E-commerce. Such frameworks may include laws and regulations for electronic documents and e-signature, electronic payments, customer protection measures such as the right of withdrawal (procedures for returning products), and privacy and data protection regulations, including safeguards for the use of personal information (the right to be forgotten) (OECD/WTO 2017). These policies can provide an adequate legal and regulatory framework that mitigates transaction risks and provides transparency within E-commerce (OECD/WTO 2017; OECD 2019c).

Given the significance of E-commerce in today’s economy and government policy in E-commerce facilitation, standardized policies regarding E-commerce are increasingly gaining the attention of governments across the world. More than 60% of regional trade agreements that entered into force between 2014 and 2016 had a chapter on E-commerce (Wolfe 2019). This also reveals the progress on this issue, since such policies were in their infancy or non-existent two decades ago (Desai et al. 2003). Empirical research also confirms the importance of government policy in the development of E-commerce. For example, Scupola (2003) showed that government policy strongly influenced the adoption of E-commerce by SMEs in southern Italy. Similarly, Cui et al. (2006) argued that government policies influenced the probability of firms entering the e-business market in China. In a multi-country study on the role of governments in firm E-commerce participation, Kraemer et al. (2006) also demonstrated that data security, inadequate legal protection, and unsupportive business laws were among regulatory barriers for firms considering adopting E-commerce.

## Review Method

We aim to identify and evaluate conceptual and empirical studies on international E-commerce policy and recommend future research avenues. To do so, we adopt a systematic analysis of relevant publications. This approach was deemed appropriate because it is a transparent process to produce a reliable and rigorous overview of extant research on a topic (Petticrew and Roberts 2008). It also ensures replicability for future research and allows examining and synthesizing relevant works (Jones et al. 2011; Tranfield et al. 2003). In our analysis, we follow the principles of transparency, clarity, focus, equality, accessibility, broad coverage, and synthesis, as Thorpe et al. (2005) suggested. Inspired by previous literature reviews in business and management journals (e.g., Jones et al. 2011; Papanastassiou et al. 2019; Pisani and Ricart 2015), we selected relevant literature by adopting the following procedure.

First, we defined our search string. Our aim was to capture publications that focused on public policy in relation to E-commerce with implications for cross-border activities. We then used a Boolean search of predetermined keywords in Thomson Reuters Web of Science. As “E-commerce policy” is a multidimensional concept that has evolved over time and relies on contributions from a wide range of disciplines and information sources, it was not viable to narrow down the review to only a few clearly defined keywords (Nippa and Reuer 2019). Rather, we chose keywords based on the evolution of our knowledge and understanding of E-commerce and relevant policies (cf. Papanastassiou et al. 2019). Thus, we decided on two sets of keywords. One related to E-commerce, and included “ecommerce,” “E-commerce,” “m-commerce,” “online sales channel,” “digitalization,” and “ibusiness”; the other related to policy, and included “policy,” “governance,” “legislation,” “law,” and “regulation.” We used the keywords in each set in combination with the keywords in the other to ensure that the articles retrieved included concepts related to both E-commerce and policy.

Our point of departure was the year 2010. Even though the E-commerce phenomenon started during the 1990s (OECD 2019c), we chose 2010 as our cut-off point because E-commerce and related policies have evolved so rapidly and altered so dramatically that earlier literature on the subject was deemed less relevant to the current reality of E-commerce. Even in the mid-2000s, E-commerce was in its infancy and traditional trade was still the norm in most countries including developed countries such as Japan and France (Kraemer et al. 2006). Since then, many countries have embraced E-commerce and accordingly designed new policies, or updated and altered existing ones. Therefore, to gain an up-to-date understanding of E-commerce and relevant policies, we used the year 2010 as the initial year to search for published works, with December 2020 as the end date. This helped us capture the evolution of E-commerce policies over the last ten years. We did not limit the results to a list of preselected top journals, as this would have reduced the coverage of relevant sources (Webster and Watson 2002). Finally, through this initial screening process, we retrieved 557 publications.

Next, we examined the publications to determine whether individual articles were in line with our inclusion/exclusion criteria. We only included publications that focused on policy designed and implemented by a government, its agents, or other official bodies (cf. Clegg 2019) in relation to E-commerce. We also ensured that the focus of the papers was on E-commerce, not ICT or other means of using digital technologies to conduct business such as digital marketing. We removed those publications from our sample that were not a complete match. We also excluded conference papers because of the variability in peer review processes (cf. Jones et al. 2011). This allowed us to narrow our database to 20 articles.

Because of this limited number of academic publications and the fact that international bodies are important policy-makers that shape regional and global E-commerce policies, we also reviewed published reports by intergovernmental bodies and organizations such as the OECD, the United Nations Conference on Trade and Development (UNCTAD), the World Economic Forum (WEF) and the WTO. Not limiting our review to the academic literature helped provide a sufficiently rich and practical understanding of the topic (Adams et al. 2017). To find relevant publications from the sources, we consulted their official websites as well as Google Scholar by using the same keywords as described above. The screening process resulted in 19 published books and reports. We further checked these sources to ensure that they were relevant and fit our inclusion criteria, and accordingly decided that 14 works were relevant (see Table 1). We also found one white paper (Lianos et al. 2019) published by the WEF that fit our inclusion criteria.

**Table 1:** Books and reports by intergovernmental bodies and organizations regarding E-commerce policy

Source, year	Publication name	Key issues discussed	Regions/countries concerned
OECD (2016b)	Consumer Protection in E-commerce: OECD Recommendation	Introducing the benefits of E-commerce as well as the complexity of the online environment and related risks for consumers	Global
OECD/WTO (2017)	Aid for Trade at a Glance 2017: Promoting Trade, Inclusiveness and Connectivity for Sustainable Development	Improving and development of E-commerce environment and policy for developing countries	Developing countries
OECD (2018)	Economic Outlook for Southeast Asia, China and India 2018: Promoting opportunities in e-commerce	A descriptive look at the opportunities and challenges regarding the rapid growth of cross-border E-commerce	Southeast Asia, China, and India
OECD (2019b)	The Role of Digital Platforms in the Collection of VAT/GST on Online Sales	Developing international standards for consistent and efficient tax systems in the context of cross-border E-commerce	Global
OECD (2019c)	Unpacking E-commerce: Business Models, Trends and Policies	Introducing several key policy areas regarding e-commerce such as consumer protection, cross-border E-commerce, taxation, and e-business models	Global
OECD (2021)	Recommendation of the Council on OECD Legal Instruments Consumer Protection in E-commerce	Discussing ways to protect E-commerce consumers in times of crisis with a focus on the COVID-19 crisis	Global
UNCTAD (2015)	Information Economy Report: Unlocking the Potential of E-commerce for Developing Countries	Examining opportunities and challenges faced by enterprises in developing countries that want to engage in E-commerce	Developing countries
UNCTAD (2018a)	Digitalization and Trade: A Holistic Policy Approach is Needed	Exploring opportunities and challenges of E-commerce and policies to unlock the potential for developing countries to benefit from E-commerce	Developing countries
UNCTAD (2018b)	Fostering Development Gains from E-commerce and Digital Platforms	Addressing the role of digital platforms for E-commerce in developing countries and the main barriers to E-commerce participation	Developing countries
UNCTAD (2019)	Rapid eTrade Readiness Assessments of Least Developed Countries: Policy Impact and Way Forward	Assessing the readiness of least developed countries to engage in E-commerce and providing a thorough overview of the E-commerce situation in such countries and recommending supportive policy measures	Least developed countries
UNCTAD (2020)	Covid-19 and E-commerce: Impact on Businesses and Policy Response	Investigating the impact of COVID-19 on E-commerce businesses with a focus on least developed countries	Least developed countries in Africa and Asia–Pacific
WEF (2019)	Africa E-commerce: Agenda Roadmap for Action	Offering a framework and refresh policies for constructive E-commerce growth in Africa	Africa
Lianos et al. (2019)	The Global Governance of Online Consumer Protection and E-commerce: Building Trust (Published by WEF)	Offering a brief overview of the online consumer protection landscape and the actors within it	Global
UN (2019)	Selected Issues in Cross-Border E-Commerce Development in Asia and the Pacific	Looking at policy and regulatory issues in cross-border E-commerce	Asia and the Pacific
Schulte-Nölke et al. (2020)	The Legal Framework for E-commerce in the Internal Market (Written at the Request of European Parliament’s Committee)	Offering a snapshot of the current EU legislative scheme that complements the E-commerce Directive; examining the need to reform the regime of the E-commerce Directive	EU

To conduct the analysis, instead of employing a deductive procedure based on a predetermined analytical framework, we followed an inductive approach of theme identification and adopted a systematic process of interpretative synthesis (cf. Jones et al. 2011; Sinkovics and Reuber 2021). We adopted principles of thematic coding from qualitative research to identify themes from our data, where the data were the publications (Bouncken et al. 2021; Jones et al. 2011; Sinkovics 2018; Thorpe et al. 2005). These themes (see Table 2) represent the main concepts and core ideas and arguments that each paper addresses.

## Discussion of Findings

**Fig. 1:**
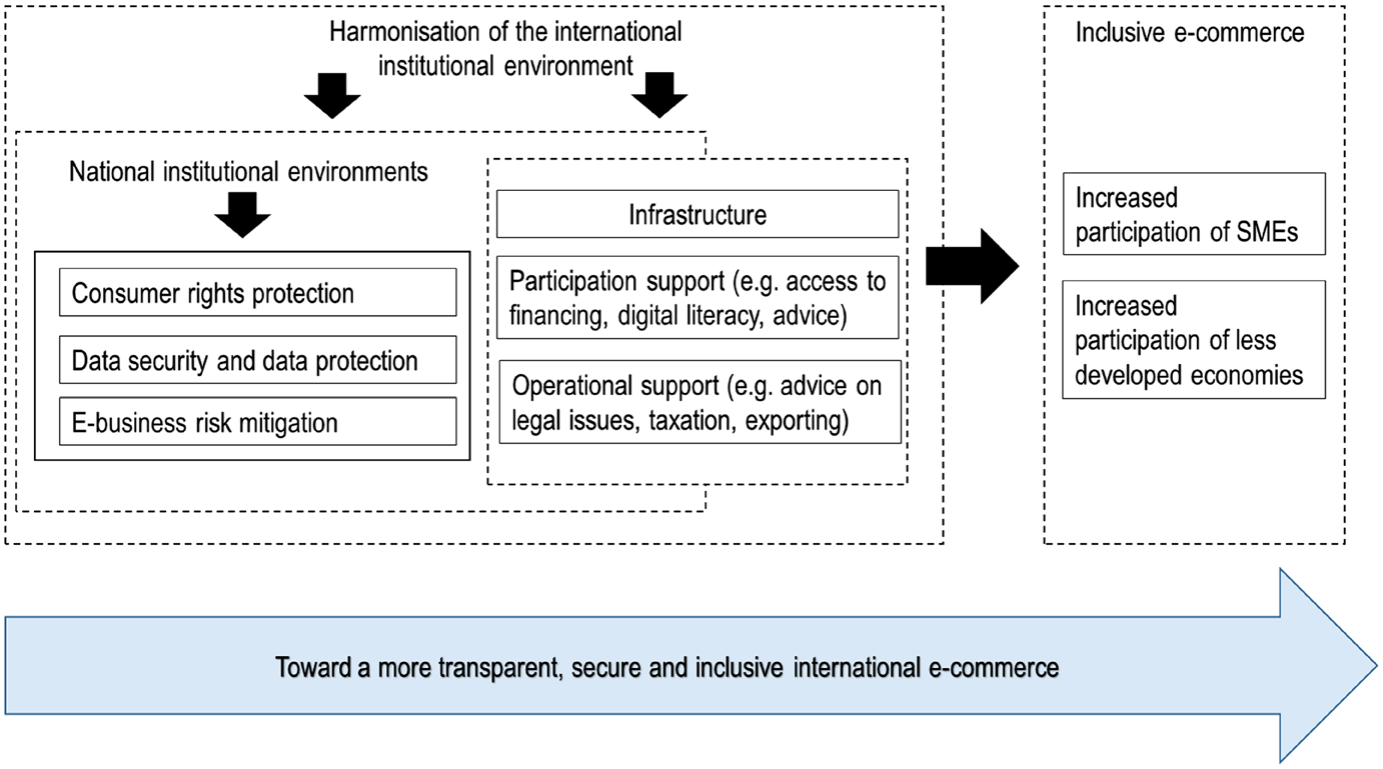
Policies for more trustful and inclusive international E-commerce

Table 2 provides an overview of the themes that emerged from our analysis. Figure 1 demonstrates how the themes relate to each other. In brief, strong formal institutions enable E-commerce adoption and promote consumer trust in this digital context (OECD/WTO 2017). This is because they provide a supportive environment, which is a prerequisite to the implementation of sound laws and regulations related to consumer, data, and privacy protection. Strong institutions and the ensuing trust in E-commerce (Lu et al. 2018) form the foundation that enables the greater participation of smaller firms and developing countries. The rest of this section discusses each theme in detail. Within each sub-section, we first provide an overview of the thematic area, followed by a summary of key recommendations derived from the publications in our sample. Table 3 at the end of the section summarizes the key points and recommendations.

**Table 2:** Themes and the relevant publications

First-order thematic area	Second-order theme description	Publications by intergovernmental bodies (in alphabetical order)	Academic publications (in alphabetical order)
Formal institutions	General institutional environment	OECD/WTO (2017); OECD (2018); UNCTAD (2015, 2019)	Froese (2019); Kardes et al. (2020); Martinez and Williams (2010); Neeraj (2019); Okoli et al. (2010); Zhu and Thatcher (2010)
	Consumer protection	Lianos et al. (2019); OECD (2019c, 2021, 2016b); OECD/WTO (2017); UN (2019); UNCTAD (2015)	Alexandru et al. (2014); Barkatullah (2018); Binczak et al. (2018); Kardes et al. (2020)
	Data protection and privacy	Lianos et al. (2019); OECD/WTO (2017); UN (2019); UNCTAD (2015)	Anic et al. (2019); Barkatullah (2018); Meltzer (2019); Neeraj (2019); Weber et al. (2020); Wolfe (2019)
Inclusiveness	E-commerce and SMEs	OECD (2019c); OECD/WTO (2017); UNCTAD (2015)	Gessner and Snodgrass (2015); Hashim (2011); Kurnia et al. (2015)
	Less-developed countries	OECD (2018); OECD/WTO (2017); UNCTAD (2015); UNCTAD (2018b, 2019, 2020); WEF (2019)	Broome (2016); Hashim (2011); Kardes et al. (2020); Ma et al. (2018); Makame et al. (2014); Martinez and Williams (2010); Okoli et al. (2010); Xiao and Zhang (2020)

**Table 3:** Summary of E-commerce policy areas, challenges, and recommendations

Policy area	Implications for E-commerce	Challenges	Policy recommendations	Possibility for empirical examination	Data source example
E-business risk mitigation	The efficiency of the formal institutional environment helps mitigate the risk of E-commerce participation	The existing formal institutions in many countries are mostly based on a traditional framework; arguably these cannot be applied to E-commerce in an efficient and effective manner	As opposed to general ICT policies, governments can introduce specific E-commerce policies concerning digital signatures, intellectual property rights for digital products, and server localization Governments can also develop cyber laws that make electronic transactions safer	Examining the relationship between the efficiency of a country’s institutional environment and E-commerce adoption (e.g., Okoli et al. 2010; Oxley and Yeung 2001)	Publications by e.g. UN (2019) and OECD/WTO (2017), and sources such as the Worldwide Governance Indicators reported by the World Bank
Consumer protection	Regulations that protect buyers in online markets increase consumer trust and participation in E-commerce	Because of no or limited real contact with sellers and inherent risks of E-commerce such as products not being delivered, consumers may be deterred from engaging in E-commerce	Governments need to ensure that online buyers are entitled to the same level of protection as in conventional transactions Intergovernmental bodies also need to address the challenge of drawing a balance between promoting global digital markets and maintaining policy space for governments to protect consumers within their borders	Looking into the relationship between public policies and laws aimed at protecting consumers and their willingness to participate in E-commerce (e.g., Binczak et al. 2018)	Publications by e.g. UN (2019) or UNCTAD (2015)
Data protection and privacy	The protection of consumers’ data is important because, first, the internet is a medium that can easily be misused by dishonest or fake sellers to exploit consumers’ data, and second, individuals are increasingly aware of the value of their data	While states define themselves as guardians of their traditional territory, cross-border E-commerce challenges this conception, as the global flow of data is unstoppable. Yet, citizens still expect states to protect their data and privacy. Consequently, designing policies regarding data protection is challenging	A global system of digital trade governance needs to be developed that includes an agenda aimed at assuring domestic regulators that the global flow of data will not undermine achieving domestic regulatory goals Governments can also adopt WTO international trade rules that provide key elements of a system of digital trade governance	Examining the relationship between privacy and data protection regulations, such as e-payment and cybersecurity laws, and consumers’ trust in E-commerce	Global Cyberlaw Tracker database reported by UNCTAD (2017)
Inclusion of SMEs in E-commerce	SMEs play an important role in the global economy, yet in both developed and less-developed countries, they have mostly been unable to leverage the advantages of E-commerce	Dealing with the logistics of customer delivery and return is usually more challenging and costly for SMEs than larger firms. The same holds for handling differences in customs and taxation and legal issues. Even in terms of simple connectivity indicators, SMEs lag behind	Governments can encourage SMEs to adopt E-commerce by providing them with human, financial, and technological resources as well as legal expertise Governments may also introduce new rules aimed at increasing transparency and fairness, helping SMEs to deal with regulatory uncertainty	Exploring whether and how past specific public policies aimed at encouraging smaller firms to participate in E-commerce have been successfull	Survey instrument to collect data from SMEs (e.g., Okoli et al. 2010); also qualitative data derived from asking SMEs about related issues (e.g., Scupola 2003)
Inclusion of less-developed countries in E-commerce	Less-developed countries lag behind developed economies in E-commerce participation. Not only do these economies benefit less from E-commerce, but E-commerce firms in developed countries also face difficulty establishing a presence in these countries	Many less-developed countries do not have the ICT infrastructure, experience, or knowledge to promote and diffuse E-commerce. Additionally, less-developed countries usually suffer from the absence of a supportive legal environment for E-commerce	Governments may design policies aimed at improving ICT infrastructure, legal frameworks, logistics and transportation, and citizens’ digital skills Promotion of cross-border exports through E-commerce by developing their own cross-border E-commerce platforms is another proposed solution An effective way forward is to create a global inclusive E-commerce adoption framework	Assessing the E-commerce readiness of less-developed countries and identifying challenges and potential solutions (e.g., Okoli et al. 2010; Oxley & Yeung 2001)	The World Bank’s *Ease of Doing Business Index* can be used to assess countries E-commerce readiness and identify challenges. Asking experts’ opinion to assess factors affecting E-commerce adoption may also be useful (e.g., Okoli et al. 2010)

### Formal Institutions

Generally, institutions can be defined as formal rules (e.g., laws and regulations) and informal constraints (e.g., norms of behavior and self-imposed codes of conduct) that enforce mechanisms that set the “rules of the game” and the boundary conditions under which business occurs (North 1990). Informal institutions are socially constructed rules that are generally considered to fall beyond government control (Clegg 2019). Given the aim of this study is to achieve a better understanding of E-commerce policy, our analysis focuses on the relevant aspects of formal institutions.

#### E-business Risk Mitigation

##### The Issues

The quality of the national institutional environment – the efficiency of the legal system and government policy – can help mitigate the risks associated with founding and operating an E-business and thus plays an important role in facilitating E-commerce adoption (Martinez and Williams 2010; OECD 2019c; UNCTAD 2015) and growth (Okoli et al. 2010). In weak institutional environments, the high regulatory uncertainty associated with inefficient or non-existent regulations related to data protection, electronic payment, intellectual property rights, and taxation may discourage business participation in E-commerce or lead to grey zones of international trade that enable tax evasion and the circulation of counterfeit products (cf. Jean and Tan 2019; OECD 2018). The protection of intellectual property rights is especially important to E-commerce as it is underpinned by digital technologies (OECD 2018). A strong regulatory environment, on the other hand, encourages participation in E-commerce because it provides a transparent legal framework in the area of electronic transactions, privacy and data protection, consumer rights protection as well as cybercrime prevention (OECD/WTO 2017; OECD 2018).

However, when governments first attempt to set rules and regulations to aid the growth of the E-commerce sector, they tend to build on past experiences and existing rules and regulations. In line with the unique nature of the digital ecosystem, this approach is likely to create a new set of challenges (cf. Neeraj 2019). Traditional frameworks that regulate trade center on the categorization of products into goods and services. However, within E-commerce, many products previously traded in a tangible form, such as books, music CDs, movie DVDs, and software CDs, are now transferred digitally through the flow of data. This raises doubts as to whether conventional regulations and laws can be applied to trade in such hybrid offerings that have elements of both goods and services. Another challenge is the current oligopolistic nature of the global electronic market, which leads to serious concerns regarding anticompetition (Neeraj 2019). For example, Amazon is the largest online retailer in the world; in 2013, it sold more online than its 12 largest competitors combined (Neeraj 2019).

##### Recommendations

To help E-commerce grow, governments are advised to introduce policies that improve the national institutional environment. This need is even more pronounced in emerging economies as they are more likely be disadvantaged by institutional voids (Agarwal and Wu 2015; Oxley and Yeung 2001) Further, governments are advised to go beyond general ICT policies and introduce specific E-commerce policies related to digital signatures, intellectual property rights for digital products, and server localization (Okoli et al. 2010; UNCTAD 2019). Such policies, in combination with a strong and supportive institutional environment including unambiguous rule of law, political stability, transparent regulatory system, and strict control of corruption, are expected to foster E-commerce adoption (Martinez and Williams 2010) and encourage E-commerce firms from developed economies to invest in less-developed countries (Coeurderoy and Murray 2008). Further, once the necessary regulations are in place, governments need to increase stakeholder awareness of such regulations (OECD 2019c). This is important, because governments may not be able to reduce regulatory uncertainty relating to E-commerce without stakeholder engagement and effective communication (OECD 2019c).

#### Consumer Protection

##### The Issues

Cross-border E-commerce has helped consumers in various ways. Specifically, it broadens the scope of available products and services and facilitates price comparison (OECD 2016a). Further, compared with conventional commerce and documents written on paper, verification techniques such as digital signatures can make electronic transactions more reliable (Barkatullah 2018). In electronic transactions, buyers usually have a digital record of what they have ordered, which they can use to claim their purchase if required.

However, through an electronic transaction, buyers have no or limited face-to-face contact with sellers. In contrast, a traditional purchase experience provides individuals with a social context and a wider range of cues to make their purchase decisions including the ability to see and touch a product and judge sellers’ professionalism (Lianos et al. 2019). Further, E-commerce may expose buyers to several risks that are less relevant in conventional commerce. For example, online shops can vanish after an order is placed and paid for, products may not be delivered, or the consumer may receive a low-quality or damaged product (Barkatullah 2018). Gaining sufficient consumer trust is particularly challenging in cross-border E-commerce because of differences in national regulations on consumer protection. In the international E-commerce space, one of the parties to the transaction may be from a jurisdiction with a less-advanced rule of law, or be unfamiliar with the other country’s legal system (Lianos et al. 2019). In such cases, consumers may perceive that they do not benefit from services such as delivery, returns, and the security of online payment to the extent they would in their home country. For example, within the EU, although electronic security is generally adequate, E-commerce consumers still tend to purchase from home suppliers (Gomez-Herrera et al. 2014). As a result, cross-border E-commerce remains largely limited to geographically close trading partners (OECD 2019c).

It is therefore not surprising that in recent years scholars have paid significant attention to the legal protection of consumers in E-commerce (e.g., Meltzer 2019; Weber et al. 2020; Wolfe 2019). Some researchers have conducted cross-country comparative studies of policies regarding consumer protection in the E-commerce context. For example, by analyzing E-commerce chapters of trade agreements, Wolfe (2019) compares the US with the EU to examine the differences in how these territories address consumer protection when engaging in E-commerce with Canada. The author concludes that governments are slowly learning the implications of cross-border E-commerce and are therefore introducing obligatory provisions regarding E-commerce consumer protection in free trade agreements. Some researchers have compared developed with less-developed countries in terms of E-commerce policy regarding consumer protection. For instance, comparing Indonesia with the EU and the US, Barkatullah (2018) argues that Indonesia is yet to implement adequate regulations concerning the protection of E-commerce consumers.

##### Recommendations

Offering consumer protection is a prerequisite to E-commerce participation. However, it is not without its challenges in the international E-commerce space. In their simplest form, consumer protection policies need to ensure that consumers buying online are entitled to the same level of protection as those engaging in conventional transactions (OECD 2019a). Policy-makers are advised to create a policy framework for electronic settlement and actively identify the barriers consumers face when engaging in E-commerce (OECD 2016a). A suggestion for good practice is the creation of national E-commerce platforms such as forums for E-commerce (OECD 2016a). These platforms can provide a neutral arena for various stakeholders – e.g., consumer groups and firms – to discuss the design and implementation of E-commerce regulations. Consumer protection policies can potentially include all stages of an E-commerce transaction (Lianos et al. 2019): the pre-purchase stage (e.g., advertising and information requirements), the purchase stage (e.g., contract term and online payment security), and the post-purchase stage (dispute resolution and redress requirements). Further, at a more global scale, intergovernmental bodies such as the WTO and the OECD need to ensure that they draw a balance between promoting global digital markets and maintaining policy space for governments to protect E-commerce consumers in their territories (Neeraj 2019).

#### Data Protection and Privacy

##### The Issues

Some authors do not draw a clear distinction between consumer protection and personal data and privacy protection (e.g., Barkatullah 2018; Lianos et al. 2019). However, while the two concepts overlap, they are not synonymous, and require different policy priorities (Lianos et al. 2019). Consumer protection requires policies on how to treat personal information. In contrast, to address data protection, policy priorities should focus on how to ensure the safety of online transactions (Lianos et al. 2019). Therefore, data protection is arguably a distinct concept.

As consumers engage in E-commerce, they share sensitive data on the internet. The protection of these data is important for at least two reasons. First, the internet can be easily used by dishonest or fake sellers to exploit consumers’ data, violate their privacy, and even steal their personal identity or credit card information (Barkatullah 2018). Second, individuals are increasingly aware of the value of their data and online privacy (Datoo 2018). Out of fear that their privacy may be violated, some individuals may even resist using the internet (Price 2020). This is not uncommon; even in some EU states, many consumers are reluctant to participate in E-commerce (Anic et al. 2019). Data breaches, online fraud, and high-profile scandals such as the Facebook–Cambridge Analytica scandal have exacerbated the situation, and it is now a growing concern both for E-commerce consumers and for businesses (UNCTAD 2015; Wolfe 2019). These make data protection in E-commerce a significant issue for both country- and regional-level policies.

However, despite attempts to address consumer data protection, states may face a territorial dilemma (Wolfe 2019). Although traditionally states are viewed as guardians of their territory, cross-border E-commerce challenges such boundaries. This is because the cross-border flow of data is hard to control, especially with the growing importance of E-commerce in modern economies. At the same time, citizens still look to the nation state for the protection of their data and privacy (Neeraj 2019; UNCTAD 2015; Wolfe 2019). As a result, many governments have introduced privacy and data protection regulations for E-commerce. For example, some countries have assigned independent regulatory bodies to oversee safe online markets to protect consumer data and enhance consumer security and trust (Lianos et al. 2019). In China, personal data protection has been an important factor not only in diffusing E-commerce through the country but also in shaping cross-border communication and cooperation with the EU (van Deursen & Kummeling 2019; Weber et al. 2020). India has also recently introduced an E-commerce policy draft that focuses on data localization (Suneja 2020). The draft recognizes citizens’ sovereign right to their data, proposing that all companies that store Indian users’ data overseas be subject to periodic audit.

##### Recommendations

Governments are advised to play an active role in the implementation of privacy regulations and data protection regimes as well as in educating the public about their rights in online transactions (Anic et al. 2019). However, there is a discernible trend of governments restricting cross-border data flows, localizing data, and restricting internet access under the aegis of data protection, cybersecurity, and the protection of local companies (Meltzer 2019). This can undermine the development of global E-commerce and its various benefits. One proposed solution to address this is to develop a global system of digital trade governance, including an agenda aimed at assuring domestic regulators that allowing data to leave their jurisdiction will not undermine the achievement of domestic regulatory goals (Meltzer 2019). To address data protection and cybersecurity concerns, governments can also adopt WTO international trade rules that provide key elements of a system of digital trade governance (Meltzer 2019). Though not without limitations, such integrated global systems provide rules that support cross-border data flows via the internet, while allowing governments to retain the regulatory flexibility to limit data flows where deemed necessary (Howse 2016; Meltzer 2019). A recent example of a relatively effective data protection regime is the EU’s General Data Protection Regulation, which requires significant data protection safeguards from online businesses (Li et al. 2019).

### Toward Inclusive E-commerce

ICT, and by extension E-commerce, play an important role in the attainment of the UN Sustainable Development Goals (SDGs) (OECD/WTO 2017). This is evidenced by their strategic integration in targets and indicators under several SDGs; examples include SDG4b and indicator 4.4.1 pertaining to quality education and upskilling related to ICT, SDG5b emphasizing the role of ICT and other enabling technologies in the empowerment of women, and SDG17 (indicators 17.6.1 and 17.8.1) highlighting the importance of global partnerships and clearly outlining the enabling function of ICT.^2^ Therefore, designing global policy that facilitates the inclusion of developing and least developed countries in the E-commerce space is considered highly important. However, this is a daunting task because of the prevailing inequalities in E-commerce participation worldwide, with a widening gap between developed and developing countries (OECD/WTO 2017). At the level of the firm, SMEs also lag behind larger firms in E-commerce adoption and in reaping related benefits (OECD/WTO 2017; OECD 2019c).

#### E-Commerce and SMEs

##### The Issues

As it is relatively easy and cost-efficient to use the internet to conduct business, even the smallest firms can participate in E-commerce (OECD 2019c). With user-friendly platforms, small firms can set up operations without the full range of traditional in-house resources such as extensive and sophisticated IT equipment, a marketing and sales department, and the expertise to establish and maintain a web presence (UNCTAD 2015). E-commerce also helps SMEs by removing the traditional geographic distance barriers and dramatically lowering information acquisition costs (Premkumar and Roberts 1999). E-commerce appears to offer benefits to firms of all sizes.

However, both in developed and less-developed economies, SMEs have been less able to leverage these advantages (OECD/WTO 2017). In 2019, E-commerce accounted for 24% of economic turnover in large firms in OECD member countries, while merely 9% of small firms engaged in E-commerce (OECD 2020). Within the majority of such countries, large firms were nearly two times more likely to engage in E-commerce (OECD 2019c). This might be the result of SMEs’ insufficient resources, which leads to a lack of investment in innovation and organizational capabilities, and in turn, makes it difficult for them to participate in E-commerce (OECD 2019a). Similarly, it is also difficult for SMEs to obtain needed legal expertise to deal with regulatory uncertainty, which hampers their relationship with larger service providers and online platforms (OECD 2019c).

Moreover, dealing with the logistics of customer delivery and return is usually more challenging and costly for SMEs than larger firms (OECD 2019c). Yet, logistics is an important part of the E-commerce value chain. To arrange delivery, SMEs tend to depend on postal operators. Therefore they are required to be able to work out different shipping and delivery options, including their cost and pricing in advance (Gessner and Snodgrass 2015). Another challenge of engaging in cross-border E-commerce for SMEs is handling differences in customs, duty regimes, and taxation, as well as insufficient resources to gain expertise on relevant legal issues (Gessner and Snodgrass 2015). Last, even in terms of simple connectivity indicators, SMEs seem to lag behind larger firms. For example, small firms are less likely to have a website or an email to communicate with business partners and consumers (OECD/WTO 2017).

##### Recommendations

Governments can support SMEs by providing them with human, financial, and technological resources that facilitate E-commerce adoption (Kurnia et al. 2015). Some of these resources include simply assistance in establishing a website or accessing e-payment services. Governments can also offer SMEs support to deal with customs, duty regimes, taxation, and other legal issues encountered in cross-border E-commerce. As the European Commission recently proposed (EC 2018), governments are advised to introduce new rules aimed at increasing transparency and fairness, thus helping SMEs to deal with regulatory uncertainty in a predictable and trusted environment. Such policy measures can help foster an inclusive business environment in which any firm, regardless of its size, can create, deliver, and capture value through E-commerce. At a global level, there is a slowly increasing number of E-commerce initiatives to support SMEs’ E-commerce adoption. For example, the WTO, the WEF and the Electronic World Trade Platform jointly launched the Enabling E-commerce Initiative, which led to a global discussion on how SMEs can leverage E-commerce (OECD 2019c).

#### E-commerce and Less-Developed Countries

##### The Issues

The availability and quality of ICT infrastructure, as well as the necessary capabilities with respect to the deployment of new technologies, play an important role in E-commerce diffusion within a country (Shih et al. 2005). The extent to which these factors are available is arguably the main driver of the E-commerce adoption gap between developed and developing countries. It appears that the digital divide (Norris 2001) flagged two decades ago persists today. In some least developed countries, even seemingly simple access to the internet remains a challenge (UNCTAD 2019; OECD/WTO 2017). Further, although ICT infrastructure and digital literacy are important factors in the growth of the E-commerce sector, these cannot explain the differences among countries in E-commerce adoption (Okoli et al. 2010; Shih et al. 2005); they need to be complemented by high-quality public policies (Oxley and Yeung 2001), as outlined in Sect. 5.1.1.

However, governments in developing and least developed countries tend to require help from international bodies with the development of such policies. Therefore, to promote E-commerce, several initiatives have been launched over the past few years to offer assistance to governments in less-developed countries. For example, eTrade for All, launched in 2016 during a UNCTAD conference, is a multi-stakeholder initiative that aims to help developing countries participate in E-commerce, thereby providing opportunities for economic development (OECD/WTO 2017). Viewing E-commerce as having a holistic nature, eTrade for All attempts to turn digital opportunities into development gains by working with a variety of stakeholders such as international organizations, regional development banks, civil society entities, and national agencies (etradeforall 2021b). The initiative covers multiple policy areas including skills development, legal and regulatory frameworks, and ICT infrastructure and services (etradeforall 2021a).

##### Recommendations

Despite past attempts, it remains a daunting task for policy-makers to frame an inclusive E-commerce adoption policy that aids less-developed economies embrace E-commerce. Policy-makers are advised to focus on the following areas when designing reforms: construction and continuous improvement of the ICT infrastructure, improvement of the E-commerce-relevant skill base of the population, support for the development of local E-commerce platforms and the facilitation of access to online payment platforms, and awareness and trust-building among the population in relation to E-commerce solutions (OECD 2018; UNCTAD 2015).

Further, governments in less-developed countries can promote cross-border trade through E-commerce by developing their own cross-border E-commerce platforms (UN 2019). Building partnerships with the private sector and collaborating with existing large platforms that can link sellers in less-developed countries to global markets can facilitate this (UN 2019). For instance, a collaboration between Alibaba and the Government of Thailand enabled Thai farmers to sell 80,000 golden pillow durians within one minute (The Nation 2018).

## Future Research

In this section, we outline four areas for future research, with the aim of stimulating discussions on E-commerce policy in international business research.

### National E-commerce Readiness

A natural first step toward formulating an inclusive and effective national E-commerce policy is assessing a country’s E-commerce readiness (OECD, 2017). Such assessments allow policy-makers to gain an in-depth understanding of national needs, strengths, and weaknesses; accordingly, they can identify opportunities and challenges in promoting and developing E-commerce and decide where improvement is needed and what supportive regulations can be introduced (UNCTAD 2019). Published reports such as UNCTAD Programme on Rapid eTrade Readiness, the UNCTAD B2C E-commerce Index, and the UNCTAD E-commerce Policy Review are good examples of tools that help evaluate the readiness of a country for E-commerce, which in turn help identify relevant policies that could foster E-commerce participation (UNCTAD 2015). However, with a few notable exceptions (Ma et al. 2019; Zhu and Thatcher 2010), academic research in this area is rare. Understanding the E-commerce readiness of a target market is relevant for firms’ active online internationalization in general (Yamin and Sinkovics 2006). By extension, it influences the extent to which internationalizing firms can rely on E-commerce as an alternative or complement to more traditional forms of market entry (Sinkovics et al. 2013).

### E-commerce Tax Policy

Tax policy challenges are now at the top of the global agenda, particularly in regards to E-commerce, where new business models include intangible assets and less visible forms of value creation (OECD 2019c). Generally, the digital economy is imposing challenges concerning the current international taxation systems, as it is not fully compatible with the tax concept that allocates jurisdictional tax claims over profit for firms based on physical presence (Ting and Gray 2019; UNCTAD 2015). The largest challenge flagged by the OECD (2019c) is that current taxation systems are still predominantly based on physical factors and traditional models. For example, in the context of international E-commerce, the location flexibility of firms can generate novel challenges in terms of tax enforcement jurisdiction (Bruce et al. 2015). Therefore, we propose that scholarly inquiries on a more effective international taxation system for cross-border E-commerce are particularly timely and of high relevance for international business research (Anderson et al. 2020; Stewart 2018).

However, excluding a few studies predominantly focusing on technical taxation issues such as the sales tax nexus (e.g., Bruce et al. 2015), academic work on tax policy in the context of cross-border E-commerce is limited. Yet, the implications of tax policies and the issues arising around their implementation and enforcement are not only relevant for firms that venture into the various forms of cross-border E-commerce; taxation also has significant societal implications that are within the remit of international business (cf. Ahen 2021; Buckley et al. 2017; Sinkovics et al. 2021a, 2021b).

### E-commerce Policy and the Natural Environment

The urgency of the need to address climate and environmental sustainability issues is increasingly recognised in policy circles as well as in scholarly publications (Hofstetter et al. 2021; Sinkovics and Archie-Acheampong 2020). However, the environmental implications of the rapid growth of global E-commerce have received limited attention in business and management studies to date. Arguably, E-commerce – especially in the context of digital products – can reduce the environmental impact of certain traditional products. It can decrease transportation use and thus reduce the pressure on physical infrastructure (OECD 2019c). Further, it can provide a more efficient supply chain, enabling firms to meet demand while keeping surplus inventory to a minimum (Kraemer et al. 2006).

However, while this may hold true in some areas, E-commerce can also lead to increased residential deliveries, which are not as efficient as professional bulk purchases (OECD 2019c). B2C E-commerce, in the context of physical products, relies heavily on packaging, shipping, and transportation – processes that can be harmful to the environment, as they often entail considerable amounts of waste and emissions (Muñoz-Villamizar et al. 2021; Schulte-Nölke et al. 2020). E-commerce also contributes to an increase in energy consumption within residential and commercial sectors (Dost and Maier 2018) and the storage of data and the electronic devices needed for E-commerce transactions create significant carbon footprints (cf. Kim et al. 2019; Renugadevi et al. 2020). The detrimental environmental impact of electronic devices after the end of their lifecycle is another area that needs to be included in discussions about E-commerce and when designing E-commerce policies (cf. Mattila et al. 2014). Therefore, how the global expansion of E-commerce affects the natural environment and what policymakers can do to mitigate these potentially adverse effects require more scholarly attention, both within and outside of international business.

### International E-commerce Policy and Micro, Small, and Medium-sized Enterprises

Although some of the reviewed publications focus on SMEs in relation to E-commerce policy, the implications of size differences across this very diverse group of firms need more attention. To date these differences are not – or only to a limited extent – taken into account (e.g., Gessner and Snodgrass 2015; OECD 2019c). For example, Kurnia et al. (2015) regard SMEs as enterprises with fewer than 50 full-time employees, while Hashim (2011) uses an employment range of 10–250 employees to define SMEs. However, micro firms (with fewer than 10 employees), small firms (with fewer than 50 employees), and medium firms (with fewer than 250 employees) differ in terms of resources they own, and this in turn shapes the investment decisions they make (Martín-Tapia et al. 2010; McCormick and Fernhaber 2018). Even compared with small firms, micro firms have a different organizational structure, financial security, and motivations (Mir 2008). Further, micro firms comprise a considerable portion of SMEs and are more likely to experience the “liabilities of smallness” and greater vulnerability to resource constraints than their larger counterparts (Hanna and Walsh 2008; McCormick and Fernhaber 2018). On the other hand, micro firms can exhibit a high level of flexibility and adaptability in their response to consumer demands (Granata et al. 2018).

Given the particularities and significance of micro firms for the global economy (McCormick and Fernhaber 2018), future research may wish to examine the suitability of current E-commerce policies for the needs of this group of firms; specifically, are current policies sufficiently enabling different groups of SMEs to participate in domestic and cross-border E-commerce or are certain groups disadvantaged by these policies? Further, comparative studies are needed to examine these questions across countries at various stages of economic development and E-commerce readiness.

## Conclusion

This study aimed to review the academic and policy literature to take stock of policy issues and recommendations in the domain of cross-border E-commerce. The findings highlight the importance of international harmonization; not just in terms of policies related to consumer protection, data protection and the mitigation of E-business risks, but also with respect to the availability and quality of digital and physical infrastructure as well as support services that foster and enable consumer and business participation. These are important factors that need to be in place if the SDGs relying on digital technologies are to be achieved including the bridging of the still prevalent digital divide. Further, as many of the technological advancements are unprecedented, collaboration at the national and international level including public–private partnerships is essential. Transparency and trust within and across national institutions are key. However, the rise of populism and increasing threat to democratic values in western economies, the challenges stemming from institutional voids in developing economies, and the high level of control in coordinated economies adds an additional layer of complexity to achieve this. Another implication of the disruptive nature of new technologies is the need to upskill the existing workforce and ensure that new generations are sufficiently trained in these technologies. Policy makers in many countries and regions are regarding digital skills as part of their education strategies (EEA 2012; Saba et al. 2021).

Finally, it is important to recognize that E-commerce can support other economic and social activities such as higher productivity, enhanced competitiveness, improved access to information, and more inclusive development (OECD/WTO 2017). Therefore, we echo the authors of the documents we examined in proposing that the objectives of E-commerce policies need to be aligned with individual country’s broader national development agendas.

## Footnotes

1 We recognize that some studies use *e-business* to refer to the same concept as *e-commerce* (e.g., Okoli et al. 2010), and some publications use the terms *e-business*, *e-trade*, and *e-commerce* interchangeably (e.g., Jean and Tan 2019; OECD/WTO 2017). For the sake of consistency and readability, we use only *e-commerce* throughout this paper.

2 https://unstats.un.org/sdgs/indicators/indicators-list/.
